# Burnout is associated with work-family conflict and gratification crisis among German resident physicians

**DOI:** 10.1186/s12909-020-02061-0

**Published:** 2020-05-08

**Authors:** Rüya Kocalevent, Hans Pinnschmidt, Susan Selch, Sarah Nehls, Juliane Meyer, Sigrid Boczor, Martin Scherer, Hendrik van den Bussche

**Affiliations:** 1 Institute and Polyclinic of Primary Medical Care, University Medical Centre Hamburg-Eppendorf, Hamburg, Germany; 2 Institute of Medical Biometry and Epidemiology, Hamburg, Germany; 3 Institute of Biochemistry and Molecular Cell Biology, Hamburg, Germany

**Keywords:** Burnout, Gratification crisis, Work-family conflict, Postgraduate training

## Abstract

**Background:**

Studies investigating the longitudinal predictive value of burnout on both effort-reward imbalance (within the working place) and work-family conflict (between work and private life) in residents are lacking. Former cross-sectional studies showed an association of effort-reward imbalance and work family conflict with an elevated burnout risk in physicians.

**Methods:**

Data acquisition was carried out within the multi-centric, longitudinal, and prospective “KarMed” study in Germany from 2009 until 2016. Yearly surveys including validated scales: the Maslach Burnout Inventory with its three subscales (emotional exhaustion, personal accomplishment, depersonalisation), the Work-Family Conflict Scale, and the Effort-Reward Imbalance Inventory. Further independent variables were gender and parental status.The analyses were based on general linear models and general linear mixed models with repeated measures designs.

**Results:**

Significant time-fixed effects were found for all three subscales of the Maslach Burnout Inventory, with gender effects on the subscales emotional exhaustion and depersonalisation. The parental status had no significant effect on burnout. All estimated means for burnout during 6 years of post-graduate training were higher when work-family conflict and gratification crisis were taken into account. Personal accomplishment increased continuously over time as well showing neither gender differences nor influences by the parental status.

**Conclusions:**

Personal accomplishments might act as a buffer compensating to some extent for the physicians’ stress experience. Given that burnout may be associated with poor patient care, there is a need to reduce burnout rates and their associated factors in resident physicians.

## Background

Burnout among medical residents is highly prevalent [1, 2] and related to self-reported suboptimal patient care practices. Besides the learning environment, high educational demands, long working hours, lack of autonomy, a high level of work-home interference, a lack of reciprocity in professional relationships and uncertainty about the future seem to play an important role in the understanding of residents’ burnout [3–5].

The time spent during postgraduate training is formative with regard to personal development in the role of a physician and quality assurance of patient care. Many physicians experience it as a stressful, burdensome period. In their daily work, they reach the limits of their ability to work under pressure and have no capacity to check learning processes [6]. Compared to senior physicians and medical management, the group of resident physicians reported significantly higher work deficits due to stress. They experienced stress to the extent that they lacked leisure time, future prospects and clear work structures. Burnout was recently included in the ICD-11 as a syndrome “conceptualised as resulting from chronic workplace stress that has not been successfully managed” [7]: It is characterised by three dimensions: feelings of exhaustion, increased mental distance from one’s job (or feelings of negativism or cynicism related to one’s job) and reduced professional efficacy.

72% of resident physicians stated that they were confronted with a high or too high workload with 41% meeting the criteria of emotional exhaustion [8]. A recent study on prevalence and predictors of burnout in USA and European residents yielded a overall prevalence rate of 40% [9].

For physicians, work-family conflict significantly correlates with burnout [10] and emotional exhaustion [11], where female residents with children experience the daily balance of work and family more challenging than their male colleagues (Raspe 2018). Work-family conflict (WFC) has generally been defined as a ‘form of interrole conflict in which the role pressures from work and family are mutually incompatible in some respect ‘[12]. Work-family conflicts seem to be prevalent in physicians [11] and residents [9] and a potential predictor in burnout [13]. Theoretical framework, for the interrelation of work-family conflict and burnout, was so far described within the conservation of resources theory assuming that burnout can be described as a state of resource depletion due to high work-family conflicts [13, 14].

These conflicts can also be related to psychological disorders, such as, for example, depression [15], where work-family conflict can be seen as an important potentially modifiable factor associated with elevated depressive symptoms in training physicians.

For preventive and treatment purposes, the current increase of female medical students generates a need for gender sensitive analysis of burnout among residents.

Gratification crises can also play an important role in the context of burnout risk [16]. The model’s central principle postulates that (a) performance or effort must be followed by (b) recognition or reward [17]. This can be in the form of appreciation, salary, job security or career advancement. These two components have a reciprocal relationship to each other. If there is no reward, the lack of reward leads to negative emotions, which can have further negative effects on self-regulation. This is also referred to as gratification crisis [17]. The resulting stress is associated with somatic and mental illnesses, such as depression, burnout, and loss of quality of life [18]. In a former study we examined the longitudinal relationships of burnout and gratification crisis during 6 years of postgraduate medical education. The amount of burnout perceived at the beginning of the postgraduate medical education was highly associated with the perceived amount of burnout at the end [19]. Gratification crisis were significant for emotional exhaustion, yet not for personal accomplishment nor depersonalization. Significant time effects were found on all 3 subscales of the MBI, with gender effects on the subscales emotional exhaustion and depersonalization. Having kids did not have significant effects, controlled for all measurement points of burnout as well as for gratification crisis. Results of the cross-sectional study by Häusler et al. (2018) showed that work-family conflicts and the experience of a gratification crisis can be seen as predictors for the development of burnout symptoms. Where the interaction term for gratification crisis on one hand and work-family conflict on the other hand was not significant The impact was major in physicians compared to other healthcare professionals [16]. However, the study population consisted only of women. Therefore, it remains unclear whether gender could be a possible confounder in the association of burnout, work-family conflict, and gratification crisis. In addition, studies investigating the longitudinal predictive value of both effort-reward imbalance (within the working place) and work-family conflict (between work and private life) for burnout risk in residents are still scarce. A longitudinal study by Hertzberg et al. (2016) investigated changes in work-home interface stress over 5 years, and their prediction of emotional exhaustion in a nationwide cohort of Norwegian doctors. A lack of reduction in work-home interface stress was reported to be an important predictor of emotional exhaustion.

This raises the following questions for the present work:

- To what extent does burnout change over five measurement points, from the first year until the sixth year of postgraduate training, taking gender and parental status into account?
- What associations do work-family conflict and gratification crises have on burnout after six years of postgraduate training, taking gender and parental status into account?

## Method

The study is part of the research project “career course of physicians in postgraduate medical education” (“Karmed”), the first and so far only multicentre prospective longitudinal study examining the careers of physicians in Germany from the start of their careers until the end of training. The study was approved by the Ethics Committee of the Medical Association of Hamburg (PV 3063).

This study examined the professional development of 1011 physicians (see Table 1), which can be seen as representative compared to federal data [20]. They were graduates of medical studies from German medical faculties in Erlangen, Gießen, Hamburg, Heidelberg, Cologne, Leipzig and Magdeburg. At the beginning of their postgraduate training (“T1”), participants were contacted with a postal survey. Attempts were made to contact 2107 persons. The set of questionnaires was administered to a sample of 1011 persons. Therefore the response rate was 48%. The participants returned the questionnaire with an anonymous code for identification in the longitudinal section as well as a personalised contact sheet separate from the questionnaire to the study centre in Hamburg (pseudonymisation). This procedure ensured the anonymity of the survey and allowed the address database to be updated throughout the survey period. All participants received €10 per completed questionnaire, and also took part in the drawing for a prize worth €350. After T1 in 2009, five further survey-measuring points followed until 2015.

**Table 1:** Sociodemographic characteristics of the study sample at baseline

	Female physicians	Male physicians	Total
Age (median)	26	27	26
Sex n (%)	665 (65.7)	346 (34.2)	1011 (99.9)
Children yes n (%)	55 (8.3)	29 (8.4)	85 (8.4)
In a relationship	458 (68.9)	240 (69.4)	699 (69.0)

**Fig. 1:**
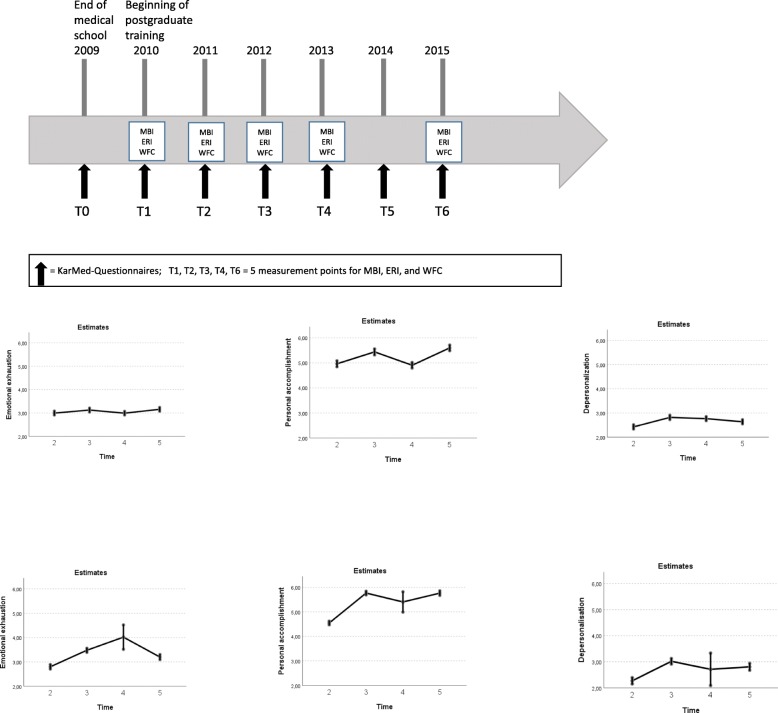
Measurement points followed by (**a**) burnout time effects without work-family conflict and gratification crises as covariates, and (**b**) burnout time effects including the continuous covariates of work-family conflict and gratification crisis

The study participants were asked to provide various pieces of personal information (sociodemographic variables), including how many children lived in the household. During five measurement points (see Fig. 1), the targeted individuals were asked to complete a survey containing a set of questionnaires, the Work-Family Conflict Scale (WFC) [12], the Effort Reward Balance Questionnaire (ERI) [21] on gratification crises as well as the Maslach Burnout Inventory (MBI) [22].

Within the Maslach Burnout Inventory burnout is a syndrome characterised by emotional exhaustion (MBI-EE: 9 items), depersonalisation (insensitive, indifferent or cynical attitude towards clients or patients) (depersonalisation, MBI-DP: 5 items) and a positive assessment of personal performance (personal accomplishments, MBI-PA: 8 items) [22]. The German version of the MBI was validated, and yielded the following norm values for low, medium and high burnout: emotional exhaustion high > = 2.87; depersonalization high > = 2.60; personal accomplishment high > = 4.87 [23]. Cronbachs alpha in the present study was .89 for emotional exhaustion, .77 for personal accomplishment, and .74 for depersonalisation.

The Effort-Reward Imbalance (ERI) questionnaire measures the relationship between effort and reward of occupational work [15]. According to the theoretical model, a balance between stress and reward is a prerequisite for job satisfaction and job-related health [12]. The reward may consist of appropriate remuneration as well as immaterial gratuities such as appreciation of the work by superiors and/or recognition by colleagues. There are two subscales: *effort* contains six items and *reward* contains ten items, both on a 4-point Lickert scale ranging from *I totally agree* to *I totally disagree*. Items were summed up for each subscale and the ratio was calculated by dividing the sum score of the effort subscale through the sum score of the reward subscale and multiplied by the correction factor 10/6 to adjust for the unequal number of items per subscale. In case of a balance of both components, the quotient claim / reward has the value ≤1. A value > 1 is defined as imbalance leading to a professional gratification crisis [24, 25]. Cronbachs alpha in the present study was .75 for the effort scale and .77 for the reward scale.

The Work and Family Conflict Scale (WFCS) was designed to evaluate a form of role conflict where the general demands of, time devoted to, and strain created by the job (family) interfere with performing work-(family-)related responsibilities [12]. Respondents rate their level of agreement to the two scales (work-family conflict and family-work conflict) consisting of the 5 items each on a 7-point Likert scale from 1 (very strongly disagree) to 7 (very strongly agree). Scores can range from 5 to 35 points. Cronbachs alpha in the present study was .92 for the work-family conflict scale and .80 for the family-work conflict scale.

### Data analysis

Statistical analyses were performed exploratory with SPSS for Windows Version 25. The significance level was set at alpha = 0.05 (two-sided). Descriptive statistics were the mean and standard deviation of continuous variables, and absolute and relative frequencies of categorical variables. The relationship between ‘time point’ and the dependent variable ‘has no kids’ was examined by binary logistic regression analysis with time point as independent variable and ‘has no kids’ as outcome variable, accounting for autocorrelation of repeated time points within the subjects ‘ID’, assuming an AR1 covariance structure. The SPSS routine GENLINMIXED was used to calculate general linear mixed models with repeated measurement design. General linear mixed models extended the linear model so that: (a) the outcome variable was linearly related to the factors and covariated via a specific link function; (b) the outcome variable could have a non-normal distribution; and (c) the observations could be correlated.

The SPSS routine GENLINMIXED was used to calculate two general linear mixed models with repeated measurement design and to describe the relationships between the temporal course of burnout until T6 and the influencing variables: time, gender, and parental status at T6, burnout at T1 as well as the current status of work-family conflicts and gratification crisis at T6. For the identification number of the participants, ID, a random effect was assumed and time within ID as measurement repetition.

## Results

### Sample

The gender distribution in the baseline survey was 66% female physicians (*N* = 665) and 34% male physicians (*N* = 346); after 5 years of further training (*N* = 304 female; *N* = 126 male). Taking into account all available measurement time periods and covariates, the total sample at the end of the postgraduate training, involved *N* = 412 physicians. The response rates, measured at the first two measurement points, were 85 and 89%, respectively (Birck et al., 2014). Each time, the response rate, measured against the previous year’s survey, was or exceeded 85%, except in the last survey, with a response rate of 77%.

The dropout rate at the end of the study was 59.2% compared to the baseline survey .

Table 1 gives an overview of the sociodemographic variables.

The proportion of women in the study cohort rose by 4.5 percentage points over the course of the survey (from the last year of medical studies until 6 years of postgraduate training). After 6 years of postgraduate training, the median age was 34 years for male and 33 years for female physicians. The proportion of respondents with children increased from 8 to 48% over the 6 years, with a large jump to T5 (T4: 29%, T5: 43%) with all pairwise comparisons being significant (*p* < 0.001).

### Burnout time effects without work-family conflict and gratification crises as covariates

#### Subscale: emotional exhaustion

There were significant time effects between all measurement points and a significant gender effect (*p* < .05) (Table 2). All estimated mean values were classified as “high” [23]. Female physicians showed significantly higher levels (M = 3.18 SD = 0.03) of emotional exhaustion over the measurement period compared to male physicians, (M = 2.95; SD = 0.05). The parental status (Female physicians: M = 3.16; SD = 0.04; Male physicians: M = 2.98; SD = 0.04) also had a significant effect (*p* < .05) taking all measurement points of the subscale emotional exhaustion of the MBI into account.

**Table 2:** Estimated marginal means over time with 95% confidence intervals for the three subscales of the Maslach Burnout Inventory

	Time	Mean	95% CI lower bound	95% CI upper bound
*Emotional exhaustion*				
	5	3.16	3.07	3.23
	4	2.99	2.90	3.07
	3	3.12	3.03	3.21
	2	2.99	2.90	3.08
*Personal accomplishment*				
	5	5.59	5.48	5.70
	4	4.90	4.79	5.01
	3	5.43	5.31	5.55
	2	4.96	4.84	5.08
*Depersonalization*				
	5	2.63	2.54	2.72
	4	2.76	2.67	2.85
	3	2.81	2.72	2.90
	2	2.42	2.33	2.51

Continuous covariates are set to the following values: T1_MBI_ee = 1.81; T1_MBI_pa = 3.89; T1_MBI_dp = 2.61;T6_MBI_ee = 3.12; T6_MBI_pa = 5.20; T6_MBI_dp = 2.61

**Table 3:** Estimated mean values over time with 95% confidence intervals for the three subscales of the Maslach Burnout Inventory under consideration of work-family conflict and gratification crisis

	Time	Mean	95% CI lower bound	95% CI upper bound
*Emotional exhaustion*				
	5	3.20	3.10	3.31
	4	4.02	3.52	4.52
	3	3.48	3.40	3.57
	2	2.80	2.71	2.90
*Personal accomplishment*				
	5	5.76	5.67	5.86
	4	5.40	4.99	5.82
	3	5.76	5.69	5.84
	2	4.55	4.47	4.62
*Depersonalization*				
	5	2.81	2.68	2.94
	4	2.71	2.09	3.33
	3	3.02	2.91	3.13
	2	2.27	2.15	2.40

Continuous covariates are set to the following values: T1_MBI_ee = 1.81; T1_MBI_pa = 3.89; T1_MBI_dp = 1.28; T1_ERI = -0.86; T6_MBI_ee = 3.18; T6_MBI_pa = 5.33; T6_MBI_dp = 2.64; T6_ERI = -0.36; T6_WFC = 3.45; T6_FWC = 0.62

The highest estimated mean value for emotional exhaustion over the measurement period was found at T5 with M = 3.16 (SD = 0.04).

#### Subscale: personal accomplishment

There were significant time effects between all measurement time periods (*p* < .05). All estimated mean values were classified as above average “high” in positive terms [23]. There was no gender effect over the time period of measurement. The parental status also showed no significant effect considering all measurement points of the subscale personal accomplishment.

The highest estimated mean value was found at T5 with M = 5.59 (SD = 0.05).

#### Subscale: depersonalisation

There were significant differences between all measurement points apart from T3 to T4. There was a significant gender effect over all measurement time periods (*p* < .05). Compared to female physicians (M = 2.49; SD = 0.04), male physicians showed significantly (*p* < .05) higher levels (M = 2.83; SD = 0.06) of depersonalisation over the measurement period. The parental status did not show any significant effects considering all measurement points of the depersonalisation subscale.

The highest estimated mean value was found at T3 with M = 2.82 (SD = 0.05).

In summary, estimated means of the three MBI subscales increased from the beginning until the end of 6 years of postgraduate training with a high variability within the measurement points.

### Burnout time effects including the continuous covariates of work-family conflict and gratification crisis

#### Subscale: emotional exhaustion

All estimated mean values were classified as “high” [23]. The highest estimated mean value was found at T4 (M = 4.02; SD = 0.26). There were significant time effects between all measurement points (*p* < .05) and a gender effect over the measurement time period (*p* < .05). Compared to male physicians (M = 3.32; SD = 0.08), female physicians (M = 3.43; SD = 0.07) showed significantly higher levels of emotional exhaustion over the measurement period. Parental status had no significant effect.

Burnout time effects, namely the estimated mean values for the subscale of emotional exhaustion, were higher when work-family conflict and gratification crisis were included as covariates. The largest difference in estimated mean values, compared to burnout time effects excluding work-family conflicts and gratification crisis, could be found at T4 (+ 1.03) (see Tables 3 and 4).

**Table 4:** Estimated marginal means for the Maslach Burnout Inventory (subscale emotional exhaustion) with CI (95%) over time

Model	Time 2	Time 3	Time 4	Time 5
Model 1 (without ERI and WFC/FWC)	2.99 (2.91–3.08)	3.12 (3.04–3.21)	2.99 (2.91–3.07)	3.16 (3.07–3.24)
Model 2 (with ERI and WFC/FWC)	2.80 (2.71–2.90)	3.48 (3.40–3.56)	4.02 (3.52–4.52)	3.20 (3.10–3.31)
Difference	−0.19	+ 0.36	+ 1.03	+ 0.04

#### Subscale: personal accomplishment

Personal accomplishment increased during postgraduate training. Estimated mean values for T3 to T5 were classified as above average, “high” in positive terms [23]. There were significant time effects between all measurement points except for T3 to T4 (*p* < .05). There was no gender effect over the time period of measurement. Parental status also had no significant effect.

Burnout time effects, namely the estimated mean values for the subscale of personal accomplishment were higher when work-family conflict and gratification crisis were included as covariates (see Table 4).

#### Subscale: depersonalisation

All estimated mean values for depersonalisation were in the upper range [23]. There were significant time effects apart from T3 to T4, T4 to T5 (*p* < .05), with a gender effect over all measurement time periods (*p* < .05). Compared to female physicians (M = 2.53; SD = 0.08), male physicians (M = 2.87; SD = 0.09) showed significantly (*p* < .05) higher levels of depersonalisation over the measurement period. Parental status had no significant effect.

Burnout time effects, namely the estimated mean values for the subscale of depersonalisation were higher when work-family conflict and gratification crisis were included as covariates (see Table 4).

## Discussion

This study was the first study prospectively investigating gender differences and parental status in burnout, and the impact of work-family conflict and gratification crisis during postgraduate training among physician residents.

Our study showed that gratification crisis and work-family conflict were positively associated with elevated burnout symptoms over the course of time with a slight decrease of burnout after 6 years of postgraduate training for both genders. Work-family conflict accounted for an additional elevation of burnout symptoms compared to the longitudinal association of burnout and gratification crisis only [19]. The present findings also correspond to the cross-sectional findings of Häusler et al. (2018), where both, gratification crisis and work-family conflict, were gradients according to burnout risk.

Furthermore, results indicated that compared to male residents, female residents’ burnout symptoms, namely emotional exhaustion, increased significantly during postgraduate training, independently of their parental status. Male residents scored higher on depersonalisation than female residents. A possible explanation for gender differences in the MBI subscales could be different coping strategies used by males and females when dealing with long-term stress. Chen et al. (2018) reported in their study on burnout and work-family conflict in Chinese doctors’, that active coping was negatively associated with depersonalisation, indicating that a passive coping style could lead to higher scores in depersonalisation [26]. Results on gender specific coping in residents are still missing.

Results of other cross-sectional studies had already shown that work-family conflict among physicians correlated strongly with burnout and increased physicians’ attrition from the workforce [13, 26–28]. Hence, results on gender effects in work-family conflict and their impact on burnout in physicians remain contradictory. A cross-sectional study, which had examined work-family conflict in a hospital showed no gender differences [29]. Results of a different recent cross-sectional study, which had investigated work-family conflict and burnout in (Chinese) physicians, indicated that male physicians exhibited higher levels of emotional exhaustion and were more affected by lower personal accomplishments [26]. A national study by Frone (2000) found no support for gender differences in work-family conflicts in general. Yet, the results showed a higher risk for psychiatric disorders in employees who reported work-family conflicts compared to those who did not. Work–home stress, perceived job demands, and colleague support were the most important predictors of life satisfaction espacially related to female doctors’ work in a 15 years longitudinal stuy in Norwegian doctors [30].

The MBI scale *personal accomplishments* increased over the measurement points of our study, independently of gender or parental status, and was even higher when gratification crisis and work-family conflict were included in the analyses compared to the longitudinal association of burnout and gratification crisis only [19]. A buffer function is conceivable here, in line with Siegrist’s intrinsic factors, the so-called personal coping strategies, such as self-efficacy, which have a compensatory effect with regard to the increasing and variable emotional exhaustion and the increasing experience of depersonalisation [21]. A study by Crum et al. has shown that DHEA (dehydroepiandrosteron) levels outweigh those of cortisol (which is associated with negative responses, such as impaired immune function and even depression) when people receive positive reinforcement before taking on a challenge., [31]. This ratio of DHEA to cortisol is called the growth index and a higher level of DHEA allows you to thrive rather than buckle under pressure.

These findings might explain why personal accomplishment in residents increased over the course of postgraduate training and might act as a buffer compensating for the resident physicians’ stress experience to some extent.

A review on physicians’ wellness by Wallace et al. (2009) points to important negative consequences of physicians’ burnout to health-care systems by affecting patient care and safety as well as physicians’ retention in terms of career satisfaction [32]. Burnout syndromes are susceptible to depressive symptoms and suicide attempts. Thus affected physicians may sometimes be unable to satisfy their patients’ demands [33]. A prospective cohort study on residents in paediatrics showed that those residents who had a burnout and were depressed had a significantly higher risk of making medication errors [34]. Yet, a recent systematic review, showed a variability in prevalence rates of burnout among physicians and differing burnout definitions [35]. The authors highlight the importance of developing a consensus definition of burnout in order to assess the effects of burnout on physicians and patient care. So far it has been difficult to link observable quality of care with burnout [36].

A cross-sectional study on a national sample of randomly-selected physicians yielded burnout as an important predictor of (lack of) career satisfaction [37].

Furthermore, the gender difference in burnout among residents may be one reason why the representation of women drops precipitously while male physicians progress up the academic ladder from medical school (51%) to full-time faculty (38%) to full professor (21%), department chair (15%), and dean (16%) [38].

Practical implications include aims at increasing the compatibility of work and private life for both gender and that residents might experience burnout but not in a clinical sense due to compensation, such as elevated personal accomplishment over the course of postgraduate training.

### Strength and limitations

The strength of the present study include the representativeness of the study sample at T1, though the drop-out analyses of the attrition needs to be awaited. Apart from subjective data, no data on objective health status data were available, so that correlations between burnout and health outcomes (including somatic outcomes) could not be the subject of this study. At this point, the need for representative longitudinal surveys should be underlined in order to gain more precise information on the causes and consequences of stress-induced processes and the associated consequences for the demands and health behaviour of physicians.

## Conclusions

Work-family conflict and gratification crisis at the end of residency trainingwere highly associated with burnout rates during 6 years of postgraduate training, especially for female residents, independently of parental status. The increase of personal accomplishments during 6 years of postgraduate training might act as a buffer compensating to some extent for the residents’ stress experience. Interventions aimed at reducing work-family conflict and gratification crisis might be an important step in reducing burnout symptoms in residents.

## Data Availability

The datasets during and/or analysed during the current study available from the corresponding author on reasonable request.

## Abbreviations

MBI: Maslach Burnout Inventory
WFC: Work-Family Conflict Scale
ERI: Effort-Reward Imbalance Questionnaire

## References

[1] Prins JT, Hoekstra-Weebers JE, Gazendam-Donofrio SM, Dillingh GS, Bakker AB, Huisman M, Jacobs B (2010). FM. vdH: burnout and engagement among resident doctors in the Netherlands: a national study. Med Educ.

[2] West CPST, Kolars JC (2011). Quality of life, burnout, educational debt, and medical knowledge among internal medicine residents. JAMA.

[3] Shanafelt TDBK, Wipf JE, Back AL (2002). Burnout and self-reported patient care in an internal medicine residency program. Ann Intern Med.

[4] Prins JTG-DS, Tubben BJ, van der Heijden FM, van de Wiel HB, Hoekstra-Weebers JE (2007). Burnout in medical residents: a review. Med Educ.

[5] Dyrbye LSTA (2016). A narrative review on burnout experienced by medical students and residents. Med Educ.

[6] Römer R, Ziegler S, Scherer M, van den Bussche H (2017). Die Berufsverlaufszufriedenheit von Assistenzärzten und -ärztinnen nach vierjähriger Weiterbildung. Z Evid Fortbild Qual Gesundh wesen.

[7] World Health Organisation. ICD-11. https://icd.who.int/en/. Accessed 5 June 2020.

[8] Biaggi P, Peter SE (2003). U: stressors, emotional exhaustion and aversion to patients in residents and chief residents - what can be done?. Swiss Med Wkly.

[9] Marchalik D, Goldman C, Carvalho F, Talso M, Lynch J, Esperto F, Pradere B, Van Besien J (2019). R. K: resident burnout in USA and European urology residents: an international concern. BJU Int.

[10] Langballe EM, Innstrand ST, Aasland OG, Falkum E (2011). The predictive value of individual factors, work-related factors, and work-home interaction on burnout in female and male physicians: a longitudinal study. Stress Health.

[11] Dyrbye LN, Shanafelt TD, Balch CM, Satele D, Sloan J, Freischlag J (2011). Relationship between work-home conflicts and burnout among American surgeons: a comparison by sex. Arch Surg.

[12] Netemeyer RGBJ, McMurrian R (1996). Development and validation of work-family conflict and family-work conflict scales. J Appl Psychol.

[13] Langballe EM, Innstrand S, Og A, Falkum E (2011). The predictive value of individual factors, work-related factors, and work-home interaction on burnout in female and male physicians. Stress Health.

[14] Hobfoll S (1989). Conservation of resources. A new attempt at conceptualizing stress. Am Psychol.

[15] Guille C, Frank E, Zhao Z, Kalmbach A, Nietert P, Mata D, Sen S (2017). Work-family conflict and the sex difference in depression among training physicians. JAMA Intern Med.

[16] Häusler NBM, Hämmig O (2018). Effort-reward Imbalance, Work-privacy Conflict, and Burnout Among Hospital Employees. J Occup Environ Med.

[17] Siegrist J (1996). Adverse health effects of high-effort/low-reward conditions. J Occup Health Psychol.

[18] Siegrist J (2011). Berufliche Gratifikationskrisen und depressive Störungen. Psychotherapeut.

[19] Kocalevent RD, Pinnschmidt H, Nehls S, Boczor S, Siegert S, Scherer M, van den Bussche H (2020). Burnout und Gratifikationskrisen im Längsschnitt bei Ärztinnen und Ärzten während der fachärztlichen Weiterbildung in Deutschland (Epub ahead of print). PPmP.

[20] Bundesamt S (2009). Bildung und Kultur. Prüfungen an Hochschulen.

[21] Siegrist JWN, Pühlhofer F, Wahrendorf M. A short generic measure of work stress in the era of globalization: effort-reward imbalance. Int Arch Occup Environ Health. 2009;82(8):1005–13. 10.1007/s00420-008-0384-3 19018554

[22] Maslach CJS (1986). Maslach Burnout Inventory. Manual.

[23] Enzmann D, Kleiber D (1989). Helferleiden stress und burnout in Psychosozialen Berufen.

[24] Siegrist J, Starke D, Chandola T, Godin I, Marmot M, Niedhammer I, Peter R (2004). The measurement of effort-reward imbalance at work: European comparisons. Soc Sci Med.

[25] van Vegchela N, de Jongea J, Bosmab H, Schaufeli W (2005). Reviewing the effort-reward imbalance model: drawing up the balance of 45 empirical studies. Soc Sci Med.

[26] Chen L, Liu J, Yang H, Ma H, Wang H, Huang Y, Cheng H, Tang D, Liu M, Luo H (2018). Work-family conflict and job burn-out among Chinese doctors: the mediating role of coping styles. Gen Psychiatr.

[27] Adam S, Györffy Z, Susanzky E (2008). Physician burnout in Hungary: a potential role for work-family conflict. J Health Psychol.

[28] Dumelow C, Littlejohns P, Griffiths S (2000). Relation between a career and family life for English hospital consultants. BMJ.

[29] Fuss I, Nübling M, Hasselhorn HM, Schwappach D, Rieger MA (2008). Working conditions and Work-Family Conflict in German hospital physicians: psychosocial and organisational predictors and consequences. BMC Public Health.

[30] Mahmood JI, Grotmol KS, Tesli M, Moum T, Andreassen O, Tyssen R (2019). Life satisfaction in Norwegian medical doctors: a 15-year longitudinal study of work-related predictors. BMC Health Serv Res.

[31] Crum A, Salovey P, Achor S (2013). Rethinking stress: the role of mindsets in determining the stress response. J Pers Soc Psychol.

[32] Wallace J, Lemaire J, Ghali W (2009). Physician wellness: a missing quality indicator. Lancet.

[33] Maslach C, Leiter M (2016). Understanding the burnout experience. World Psychiatry.

[34] Fahrenkopf A, Sectish T, Barger L, Sharek P, Lewin D, Chiang V, Edwards S, Wiedermann B, Landrigan C (2008). Rates of medication errors among depressed and burnt out residents: prospective cohort study. BMJ.

[35] Rotenstein L, Torre M, Ramos M, Rosales R, Guille C, Sen S, Mata D (2018). Prevalence of burnout among physicians. JAMA.

[36] Angerer P, Weigl M. Physicians work conditions and quality of care: a literature review. Professions Professionalism. 2015;5(1):1–20.

[37] Keeton K, Fenner DE, Johnson TR, Hayward RA (2007). Predictors of physician career satisfaction, work-life balance, and burnout. Obstet Gynecol.

[38] Carnes M, Morissey C, Geller S (2008). Women's health and women's leadership in academic medicine. J Women's Health.

